# Loss of Pax3 causes reduction of melanocytes in the developing mouse cochlea

**DOI:** 10.1038/s41598-024-52629-9

**Published:** 2024-01-26

**Authors:** Tomokatsu Udagawa, Erisa Takahashi, Norifumi Tatsumi, Hideki Mutai, Hiroki Saijo, Yuko Kondo, Patrick J. Atkinson, Tatsuo Matsunaga, Mamoru Yoshikawa, Hiromi Kojima, Masataka Okabe, Alan G. Cheng

**Affiliations:** 1 Department of Otorhinolaryngology, The Jikei University School of Medicine, 3-25-8 Nishi-Shimbashi, Minato-ku, Tokyo, 105-8461 Japan; 2 Department of Anatomy, The Jikei University School of Medicine, Tokyo, Japan; 3 Department of Otorhinolaryngology, Toho University School of Medicine, Tokyo, Japan; 4 Division Hearing and Balance Research, National Institute of Sensory Organs, NHO Tokyo Medical Center, Tokyo, Japan; 5 Department of Otolaryngology-Head and Neck Surgery, Stanford University School of Medicine, Stanford, CA 94305 USA

**Keywords:** Developmental biology, Molecular biology, Neuroscience, Diseases

## Abstract

Cochlear melanocytes are intermediate cells in the stria vascularis that generate endocochlear potentials required for auditory function. Human *PAX3* mutations cause Waardenburg syndrome and abnormalities of skin and retinal melanocytes, manifested as congenital hearing loss (~ 70%) and hypopigmentation of skin, hair and eyes. However, the underlying mechanism of hearing loss remains unclear. Cochlear melanocytes in the stria vascularis originated from *Pax3*-traced melanoblasts and *Plp1*-traced Schwann cell precursors, both of which derive from neural crest cells. Here, using a *Pax3-Cre* knock-in mouse that allows lineage tracing of *Pax3*-expressing cells and disruption of *Pax3*, we found that Pax3 deficiency causes foreshortened cochlea, malformed vestibular apparatus, and neural tube defects. Lineage tracing and in situ hybridization show that *Pax3*^+^ derivatives contribute to S100^+^, *Kir4.1*^+^ and *Dct*^+^ melanocytes (intermediate cells) in the developing stria vascularis, all of which are significantly diminished in *Pax3* mutant animals. Taken together, these results suggest that *Pax3* is required for the development of neural crest cell-derived cochlear melanocytes, whose absence may contribute to congenital hearing loss of Waardenburg syndrome in humans.

## Introduction

*Pax3* regulates induction and differentiation from neural crest cells which are critical in the development of many organs such as eye, hair, skin, neural tube, cranium, face and inner ear^1–4^. During embryonic development, *Pax3*^+^ neuroepithelial cells including neural crest cells migrate into the otic vesicle, which is primordium of the inner ear, and give rise to melanocytes and glial cells in the developing cochlea^5–8^.

Heterozygous mutations in human *PAX3* gene cause type 1 Waardenburg syndrome, which is characterized by telecanthus (widely spaced eyes), heterochromia iridis, patchy pigmentation of hair and skin, and profound sensorineural hearing loss^9–12^. Heterozygous *PAX3* mutations also cause type 3 Waardenburg syndrome, which consists of upper limb abnormalities with type 1 Waardenburg syndrome phenotypes^9,13,14^. There are also reports of homozygous *PAX3* mutations among type 3 Waardenburg syndrome patients^15–17^. Thus, both heterozygous and homozygous *PAX3* mutations are associated with Waardenburg syndrome.

Sensorineural hearing loss is estimated to occur in 71% of Waardenburg syndrome patients^18^. It has been postulated that congenital hearing loss in Waardenburg syndrome results from developmental defects of cochlear melanocytes, which are located as intermediate cells in the stria vascularis and necessary for generating the endocochlear potential required for auditory function^6,19–22^. Although radiographic imaging of temporal bone of patients with *PAX3* mutations shows normal inner ear structure, one *PAX3* mutant mouse (*Sp*^*2H*^/*Sp*^*2H*^) has been reported to display a complete absence of cochlear components such as the organ of Corti or stria vascularis^23,24^, suggesting that the phenotype of Pax3-deficient mice is more severe than that of *Pax3*-heterozygous patients.

Pax3 deficiency has been postulated to prevent development of *Pax3*^+^ derivatives including intermediate cells in the developing stria vascularis^6^. Recently, fate-mapping experiments showed that intermediate cells have dual embryonic origins: melanoblasts and Schwann cell precursors, which are derived from neural crest cells and begin to migrate into the stria vascularis around embryonic day 15.5 (E15.5) first in the base and proceeds towards the apex^8^.

In this study, we examined *Pax3-Cre* knock-in mice^25^ which allows fate-mapping of *Pax3* expressing cells and ablation of the *Pax3* gene, and found that complete loss of Pax3 prevents formation of melanocytes (intermediate cells) in the developing cochlea. We analyzed cochleae at E18.5 as *Pax3*^*Cre/Cre*^ homozygous mice were perinatally lethal^25^. At E18.5, *Pax3* knockout cochleae showed 2 major phenotypes: 57% of animals showed smaller cochleae with normal organization of the organ of Corti, whereas 43% showed severely shortened cochlea in addition to gross organ dysgenesis such as exencephaly. Mice of both phenotypes lacked Pax3 protein expression. Furthermore, with fate-mapping and immunostaining for cochlear melanocytes, we uncovered that S100^+^ Pax3^Cre^-EGFP^+^ intermediate cells were diminished in the stria vascularis of E18.5 *Pax3* knockout cochleae, with remaining intermediate cells continuing to differentiate into *Dct*^+^ or *Kir4.1*^+^ melanocytes. Our results suggest that loss of *Pax3* leads to reduction of cochlear melanocytes, which may contribute to congenital hearing loss in Waardenburg syndrome.

## Results

### Auditory function and cytoarchitecture of the ***Pax3***^***Cre/***+^ mice

**Figure 1:**
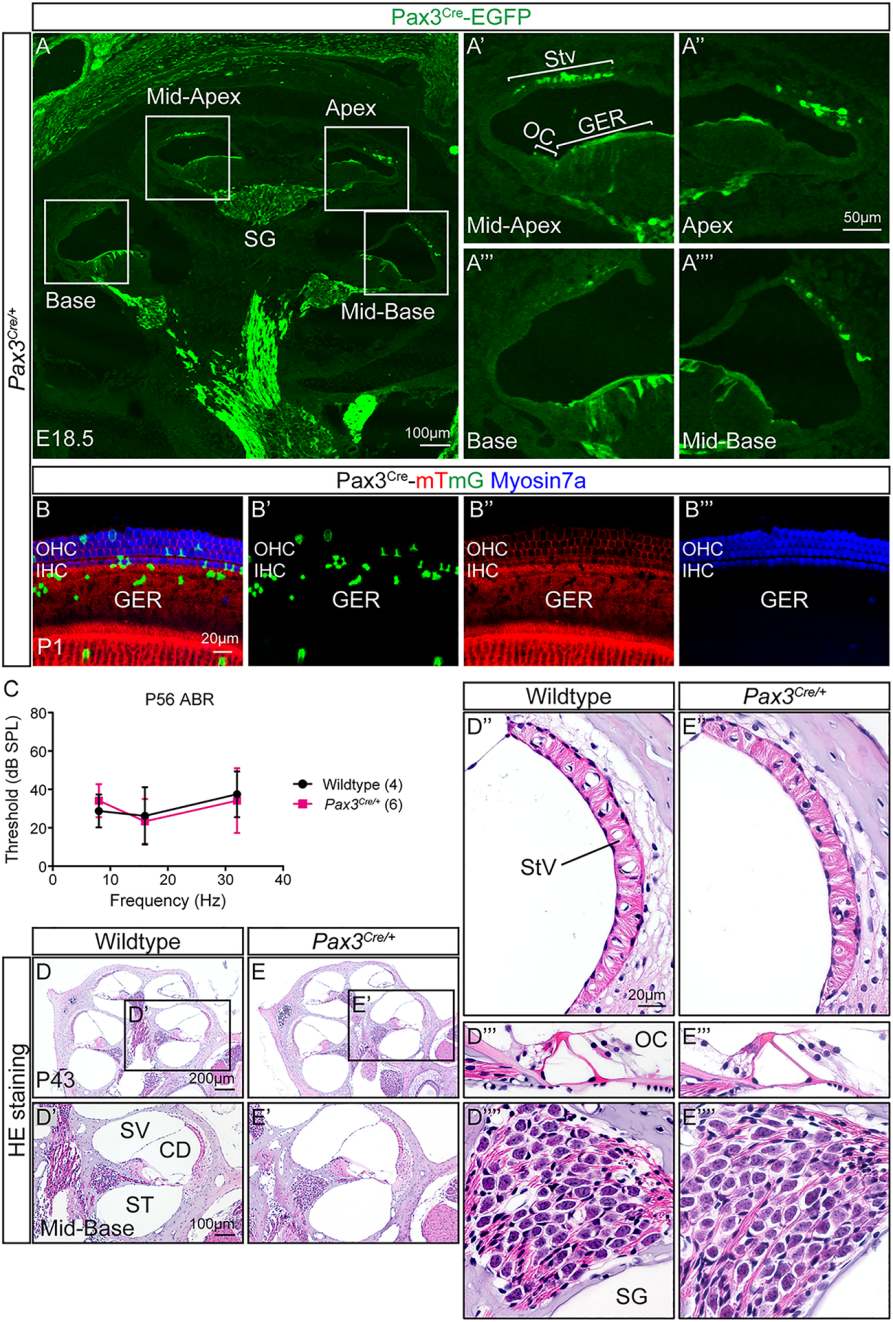
Auditory and cochlear properties of *Pax3*^*Cre/*+^ heterozygous mice. (**A–Aʹʹʹʹ**) In the E18.5 *Pax3*^*Cre/*+^ cochlea, Pax3^Cre^-EGFP^+^ cells distributed in the stria vascularis, glial cell region including the spiral ganglion, GER and organ of Corti. (**B**) Some Pax3^Cre^-EGFP^+^ cells scattered in the GER and organ of Corti at P1. (**C**) P56 *Pax3*^*Cre/*+^ heterozygous mice had no significant difference in auditory brain responses thresholds at all frequencies compared with wildtype. (**D,E**) P43 cochlea showed no difference in the stria vascularis, organ of Corti or spiral ganglion neuron between wildtype and *Pax3*^*Cre/*+^ heterozygous mouse. *ABR* auditory brain responses, *CD* cochlear duct, *GER* greater epithelial ridge, *IHC* inner hair cell, *SV* scala vestibuli, *ST* scala tympani, *StV* stria vascularis, *OC* organ of Corti, *OHC* outer hair cell, *SG* spiral ganglion; data represent mean ± S.D. (two-way ANOVA with Sidak’s multiple comparisons test). n = 4–6.

To determine the cell fate of *Pax3*^+^ derivatives in the cochlea, we used a lineage tracing approach (*Pax3-Cre; CAG-CAT-EGFP or Rosa-mTmG*) where *Pax3-*traced cells are labeled with EGFP. This approach has been used previously to fate-map derivatives of *Pax3*^+^ neuroepithelial cells including neural crest cells in various organs including the cochlea^5,6,25,26^. We first confirmed that Pax3^Cre^-EGFP^+^ cells were distributed primarily in the stria vascularis and modiolar regions, while occasionally EGFP^+^ cells were detected in the organ of Corti and greater epithelial ridge (GER) region in the E18.5 and P1 *Pax3*^*Cre/*+^ heterozygous cochleae (Fig. 1A,B). We also confirmed that *Pax3*^*Cre/*+^ heterozygous embryos had normal cochlear development (Fig. 1A), consistent with prior reports^5,6^. Previously, *Pax3* heterozygous mice (splotch mice, *Sp*) show normal hearing even though they have patchy pigmentation of skin hair like human Waardenburg syndrome patients^27^. We further assessed auditory responses of adult P56 *Pax3*^*Cre/*+^ heterozygous mice with patchy pigmentation of skin hair and similarly found that they had auditory brain responses comparable to wildtype littermates at all frequencies tested (8, 16, 32 kHz) (Fig. 1C).

Like wildtype cochlea, the P43 *Pax3*^*Cre/*+^ heterozygous cochlea displayed normal cytoarchitecture of the stria vascularis, organ of Corti and spiral ganglion (Fig. 1D,E). These data indicate that *Pax3*^*Cre/*+^ heterozygous cochlea is grossly normal and may serve as a control for *Pax3*^*Cre/Cre*^ homozygous cochlea, and also as an excellent model to examine the *Pax3*^+^ derived cellular populations in the cochlea.

### *Pax3* knockout embryos show cochlear and vestibular defects in the late embryonic period

During normal development, the otic vesicle rapidly expands and gives rise to the cochlear duct and vestibular apparatus shortly after E10.5^28–30^. Previously, *Pax3*^*Cre/Cre*^ homozygous embryos have been shown to display several developmental anomalies including loop tail and spina bifida with complete loss of Pax3 protein^25^. The degrees of anomaly are classified as mild or severe, with the latter showing exencephaly and shortened cochlea^5,6^. In this current study, Sox2 was expressed in the neuroepithelial layer of both *Pax3*^*Cre/*+^ and *Pax3*^*Cre/Cre*^ brain (Supplementary Fig. S1A–C), and Pax3^Cre^-EGFP^+^ cells were found in the neural tube and roof plate of E11.5 *Pax3*^*Cre/*+^ and *Pax3*^*Cre/Cre*^ mice with a mild phenotype (Supplementary Fig. S1A,B). Pax3^Cre^-EGFP^+^ cells were located in the lateral neural tube region of E11.5 *Pax3*^*Cre/Cre*^ mice with a severe phenotype including exencephaly (Supplementary Fig. S1C). At E11.5, *Pax3*^*Cre/*+^ heterozygous mice expressed Pax3 in both Sox2^+^ and Pax3^Cre^-EGFP^+^ neural tube cells and roof plate cells (Supplementary Fig. S1A). In contrast, Pax3 expression was not detected in either Sox2^+^ neural tube cells or roof plate cells in E11.5 *Pax3*^*Cre/Cre*^ homozygous mice with a mild or severe phenotype, indicating that Pax3 was effectively ablated regardless of the severity of the phenotype (Supplementary Fig. S1B,C). *Pax3*^*Cre/Cre*^ homozygous embryos also die by P0^25^. Thus, we characterized the morphology of embryos shortly before birth at E18.5 (27 wildtype, 37 *Pax3*^*Cre/*+^ heterozygous, 14 *Pax3*^*Cre/Cre*^ homozygous embryos from 7 litters) (Supplementary Fig. S2A–D, Table S1). We found that 8 *Pax3*^*Cre/Cre*^ homozygous embryos displayed mild anomalies (mostly loop tail and spina bifida) (Supplementary Fig. S2C, Table S1), and 6 were severe (visibly smaller, displayed exencephaly, loop tail, and spina bifida) (Supplementary Fig. S2D, Table S1).

**Figure 2:**
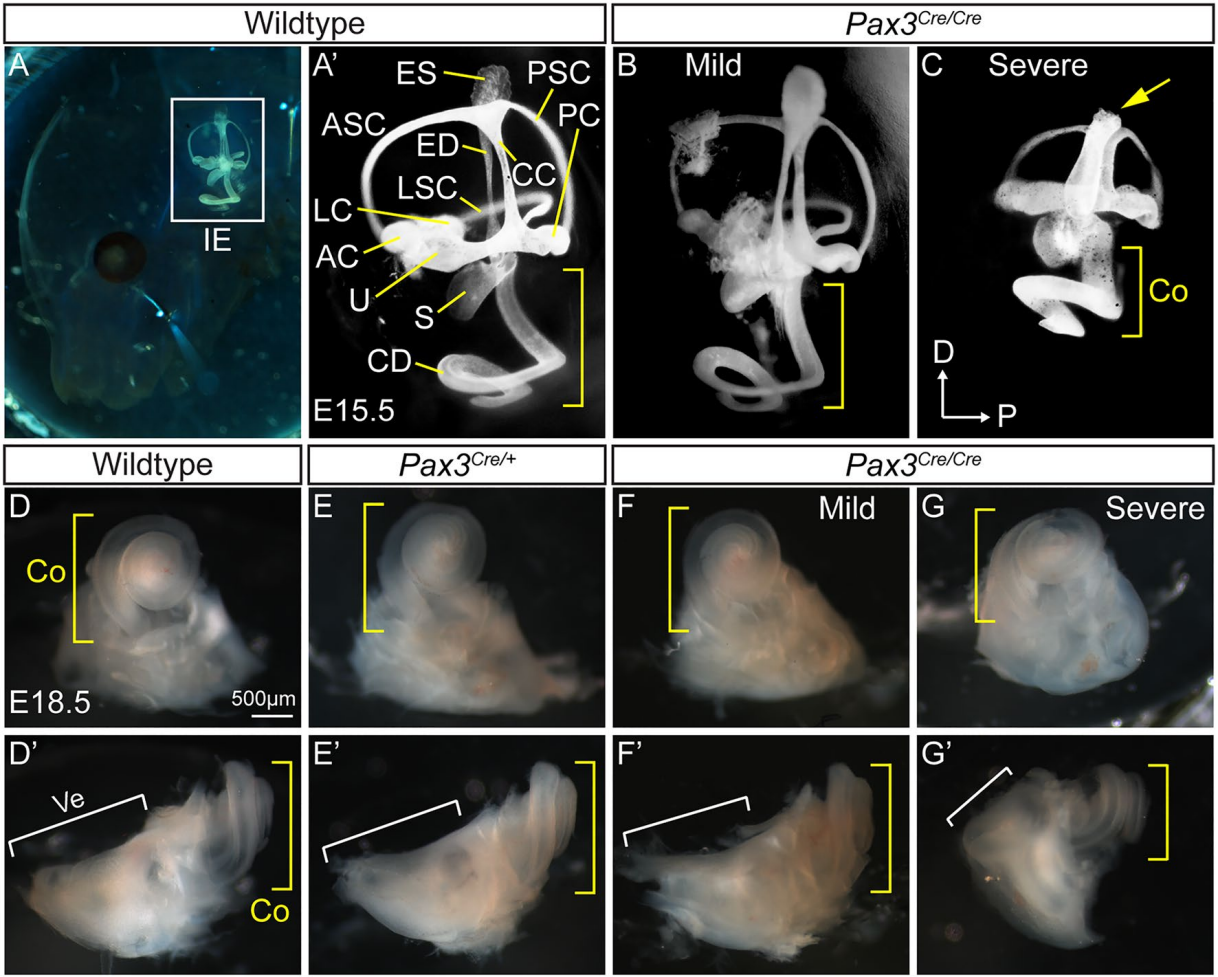
*Pax3* knockout inner ear phenotypes. (**A,Aʹ**) White latex paint was injected into the endolymph duct in the E15.5 wildtype mouse embryo. (**B,C**) Two pattern shapes (mild and severe) of the endolymph duct in the E15.5 *Pax3* knockout cochlea. Compared with two types of *Pax3* knockout inner ear, severe phenotype displayed shortened cochlear duct (yellow bracket) and semicircular canals, and diminished endolymphatic sac (yellow arrow) than mild phenotype. (**D–G**) Compared with other E18.5 samples, severe phenotype of the E18.5 *Pax3*^*Cre/Cre*^ homozygous inner ear showed smaller cochlear (yellow bracket) and vestibular organs (white bracket). *IE* inner ear, *CD* cochlear duct, *U* utricle, *S* saccule, *AC* anterior crista, *PC* posterior crista, *LC* lateral crista, *ASC* anterior semicircular canal, *PSC* posterior semicircular canal, *LSC* lateral semicircular canal, *CC* common crus, *ED* endolymphatic duct, *ES* endolymphatic sac, *D* dorsal, *P* posterior, *Co* cochlear organ, *Ve* vestibular organs.

To further examine the morphology of the inner ear of *Pax3*^*Cre/Cre*^ homozygous mice, we performed paint-fill of E15.5 cochleae, using wildtype as controls. The inner ear of E15.5 *Pax3*^*Cre/Cre*^ homozygous mice with a mild phenotype was smaller but exhibited similar morphology to wildtype control (Fig. 2A,B). However, *Pax3*^*Cre/Cre*^ homozygous embryo with a severe phenotype showed several malformations of both vestibular and cochlear organs, including underdeveloped semicircular canals, vestigial endolymphatic duct, and foreshortened cochlear duct (Fig. 2C). Similarly, at E18.5, *Pax3*^*Cre/Cre*^ homozygous inner ear with severe phenotype was noticeably smaller than wildtype, the *Pax3*^*Cre/*+^ heterozygous inner ear and *Pax3*^*Cre/Cre*^ homozygous inner ear with mild phenotype (Fig. 2D–G). These results indicate that development of the inner ear is grossly normal in *Pax3*^*Cre/Cre*^ homozygous embryo with mild phenotype but is dramatically perturbed in *Pax3*^*Cre/Cre*^ homozygous embryo with the more generalized severe phenotype.

### Some *Pax3*^+^ derivatives distribute as the intermediate cells in the stria vascularis of *Pax3* knockout cochleae

Lineage tracing experiments using *Wnt1-Cre*, *Plp1-Cre* and *Pax3-Cre* mice demonstrate that neural crest cells migrate and develop as glial cells including Schwann cells and satellite cells in the spiral ganglion and cochlear melanocytes (intermediate cells) in the stria vascularis^5,6,8^.

**Figure 3:**
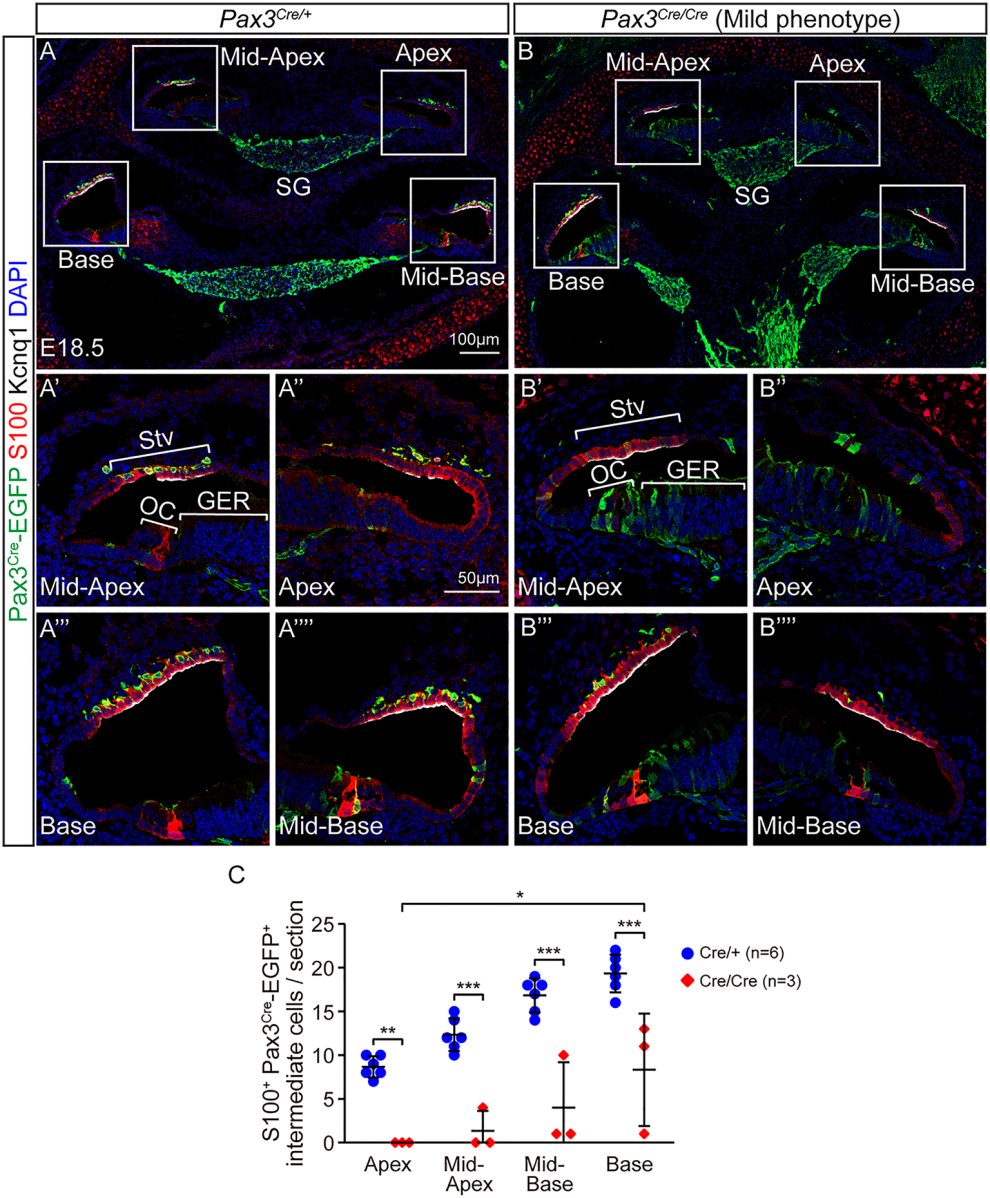
Fewer intermediate cells derived from *Pax3*^+^ derivatives in the *Pax3* knockout cochleae with a mild phenotype. (**A–Aʹʹʹʹ**) In the E18.5 *Pax3*^*Cre/*+^ control cochlea, S100^+^ Pax3^Cre^-EGFP^+^ intermediate cells were localized besides Kcnq1^+^ S100^+^ marginal cells in the stria vascularis of all cochlear turns. (**B–Bʹʹʹʹ**) In the stria vascularis of the E18.5 *Pax3*^*Cre/Cre*^ mild phenotype cochlea, some S100^+^ Pax3^Cre^-EGFP^+^ intermediate cells were detected next to Kcnq1^+^ S100^+^ marginal cells. (**C**) Quantification of S100^+^ Pax3^Cre^-EGFP^+^ intermediate cells in the stria vascularis showed significant reductions of all turns in the E18.5 *Pax3*^*Cre/Cre*^ mild phenotype homozygous embryos compared with *Pax3*^*Cre/*+^ heterozygous embryos. The basal turn contained significantly more S100^+^ Pax3^Cre^-EGFP^+^ intermediate cells than the apical turn in the stria vascularis of the E18.5 *Pax3*^*Cre/Cre*^ mild phenotype homozygous embryos. *StV* stria vascularis, *OC* organ of Corti, *SG* spiral ganglion, *GER* greater epithelial ridge; data represent mean ± S.D. *p < 0.05, **p < 0.01, ***p < 0.001 (two-way ANOVA with Tukey’s multiple comparisons test). n = 3–6.

We first determined the cellular contribution of the *Pax3*^+^ lineage in the spiral ganglion region. At E18.5, we found Pax3^Cre^-EGFP^+^ cells populating the spiral ganglion regions in the *Pax3*^*Cre/*+^ heterozygous cochleae (Figs. 1A, 3A), consistent with previous reports^5,6^. At E18.5, the *Pax3*^*Cre/Cre*^ homozygous cochleae appeared smaller than the *Pax3*^*Cre/*+^ heterozygous cochleae, although Pax3^Cre^-EGFP^+^ cells remained present in the spiral ganglion regions throughout the cochlea in both mild and severe homozygous animals (Figs. 1A, 3A,B, Supplementary Figs. S3A,B, S4A).

The mature stria vascularis has three cell types: marginal cells, basal cells and intermediate cells, the latter of which are melanocytes. By contrast, the embryonic stria vascularis is composed of only marginal cells and intermediate cells^31^. Previously, *Pax3*^*Cre/Cre*^ homozygous cochlea has been shown to display a complete loss of *Pax3*^+^ derivatives and *Dct*^+^ cochlear melanocytes in the stria vascularis at E15.5^6^. As melanocytes originate from both melanoblasts and Schwann cell precursors^8^, we hypothesize that Pax3 deficiency leads to a partial, and not complete, loss of cochlear melanocytes in the late embryonic period. First, we analyzed the distribution of the *Pax3*^+^ derivatives in the stria vascularis of the E18.5 *Pax3*^*Cre/Cre*^ homozygous cochlea. Kcnq1 is a marker for the marginal cells, and S100 marks both the marginal cells and intermediate cells in the stria vascularis^31,32^. In the *Pax3*^*Cre/*+^ heterozygous cochlea, many S100^+^ Pax3^Cre^-EGFP^+^ intermediate cells were detected next to Kcnq1^+^ S100^+^ marginal cells in the stria vascularis in all cochlear turns (Fig. 3A). By contrast, in the E18.5 *Pax3*^*Cre/Cre*^ homozygous cochlea with a mild and severe phenotype, rare or no S100^+^ Pax3^Cre^-EGFP^+^ intermediate cells were detected in the stria vascularis of the apical (Fig. 3Bʹʹ, Supplementary Fig. S4Aʹ), middle (Fig. 3Bʹ, Bʹʹʹʹ, Supplementary Fig. S4Aʹʹ) and basal turns (Fig. 3Bʹʹʹ, Supplementary Fig. S4Aʹʹʹ).

**Figure 4:**
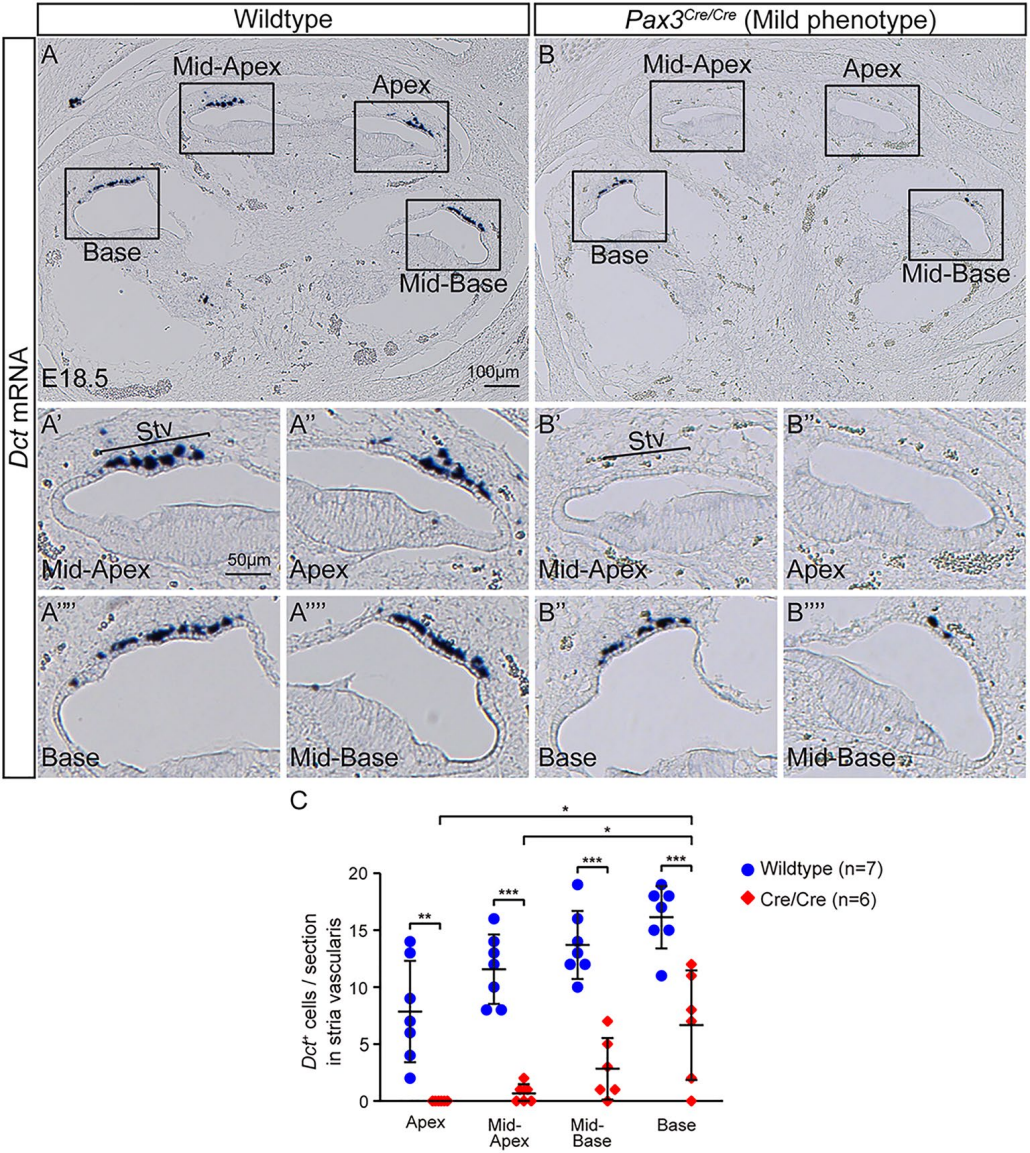
Distribution of melanocytes in the *Pax3* knockout cochleae at late embryonic day. (**A–Aʹʹʹʹ**) In the E18.5 *Pax3*^*Cre/*+^ control cochlea, *Dct*^+^ melanocytes were detected on a straight line along with the cochlear duct in the stria vascularis of all cochlear turns. (**B–Bʹʹʹʹ**) In the stria vascularis of the E18.5 *Pax3*^*Cre/Cre*^ mild phenotype cochlea, no *Dct*^+^ melanocytes were found in the apical or mid-apical turn although a few *Dct*^+^ melanocytes were detected in the mid-basal or basal turn. (**C**) *Dct*^+^ melanocytes in stria vascularis were significantly fewer in all turns of the E18.5 *Pax3*^*Cre/Cre*^ mild phenotype homozygous cochleae than E18.5 wildtype cochleae. The basal turn had significantly more *Dct*^+^ melanocytes than the apical and mid-apical turns in the E18.5 *Pax3*^*Cre/Cre*^ mild phenotype homozygous embryos. *StV* stria vascularis; data represent mean ± S.D. *p < 0.05, **p < 0.01, ***p < 0.001 (two-way ANOVA with Tukey’s multiple comparisons test). n = 6–7.

**Figure 5:**
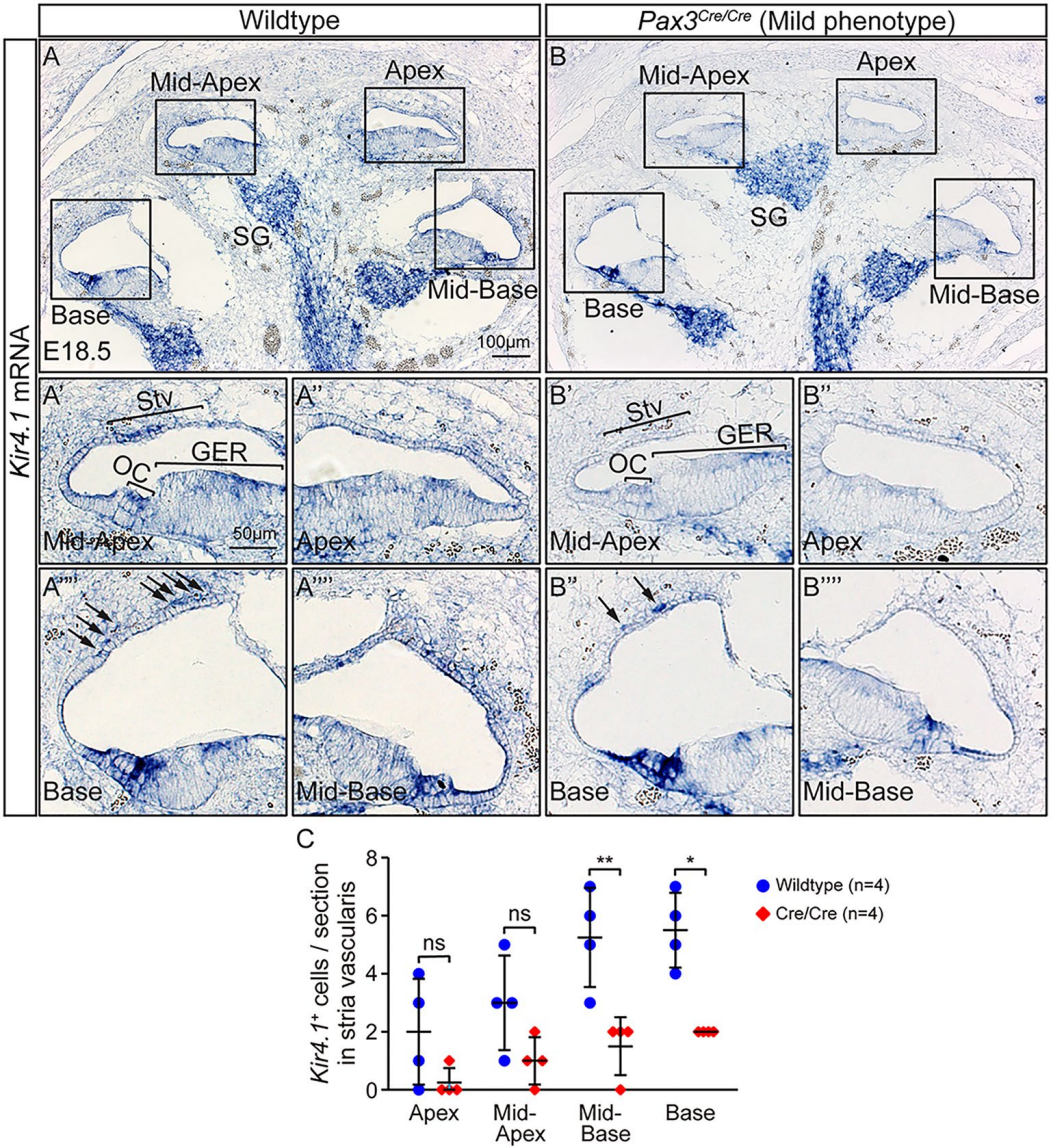
*Pax3* knockout cochleae exhibit some differentiated intermediate cells in the stria vascularis in late embryonic age. (**A,B**) A few *Kir4.1*^+^ intermediate cells (arrows) were found in the stria vascularis of each turn of the E18.5 *Pax3*^*Cre/*+^ control and *Pax3*^*Cre/Cre*^ mild phenotype cochlea. (**C**) Quantification showed significant reductions of *Kir4.1*^+^ melanocytes in the stria vascularis of the mid-basal and basal turns in the E18.5 *Pax3*^*Cre/Cre*^ mild phenotype homozygous embryos compared with the E18.5 *Pax3*^*Cre/*+^ heterozygous embryos. *StV* stria vascularis, *OC* organ of Corti, *SG* spiral ganglion, *GER* greater epithelial ridge; data represent mean ± S.D. *p < 0.05, **p < 0.01 (two-way ANOVA with Tukey’s multiple comparisons test). n = 4.

Next, we quantified intermediate cells and compared the *Pax3*^*Cre/Cre*^ homozygous (mild phenotype) cochlea with that of the *Pax3*^*Cre/*+^ heterozygous or wildtype cochlea at E18.5. All of them displayed four cochlear regions (apex, mid-apex, mid-base and base) in cross-section (Figs. 1A, 3A,B, 4A,B, 5A,B, Supplementary Fig. S3A). In each cochlear turn, there were noticeably fewer S100^+^ Pax3^Cre^-EGFP^+^ intermediate cells in the stria vascularis of *Pax3*^*Cre/Cre*^ homozygous embryos with mild phenotype than *Pax3*^*Cre/*+^ heterozygous embryos. The reduction is most dramatic in the apical turn relative to the base (Fig. 3C). Collectively, these data suggest that loss of *Pax*3 prevents normal development of intermediate cells in the cochlea, with the apical and middle cochlear turns more severely affected than the base.

### Characterizing melanocytes in the stria vascularis in the *Pax3* knockout embryos

To further characterize whether cochlear melanocytes were perturbed in *Pax3*^*Cre/Cre*^ homozygous mouse, we performed in situ hybridization for markers of melanocytes. *Dct*, a classical marker of melanocytes, was detected in all three turns in the E18.5 wildtype cochlea (Fig. 4A). In the *Pax3*^*Cre/Cre*^ homozygous cochlea (mild phenotype), we discovered markedly fewer *Dct*^+^ melanocytes in all turns, with the greatest reduction observed in the apical turn (Fig. 4B,C).

Moreover, we examined expression of the inwardly rectifying potassium channel *Kir4.1*, whose expression in the stria vascularis is crucial for development of the endocochlear potential after P7^19,20,33^. Because *Pax3*^*Cre/Cre*^ homozygous mice are lethal perinatally, we investigated *Kir4.1* mRNA expression in the E18.5 cochleae^25^. *Kir4.1* mRNA was detected in the stria vascularis of both control and the *Pax3*^*Cre/Cre*^ homozygous mild phenotype cochlea. *Kir4.1* mRNA was also detected in the organ of Corti and spiral ganglion (Fig. 5A,B). In the *Pax3*^*Cre/Cre*^ homozygous embryos with mild phenotype, there were significantly fewer *Kir4.1*^+^ cells in the stria vascularis in the mid-basal and basal turns than those in *Pax3*^*Cre/*+^ heterozygous embryos, although the apical and mid-apical turns showed no significant reduction in the number of *Kir4.1*^+^ cells between those groups (Fig. 5C). Together, these data reveal that Pax3 deficiency perturbs development of cochlear melanocytes.

## Discussion

Waardenburg syndrome is characterized by hearing loss and developmental abnormalities of melanocytes^12,34^. Genetic studies suggest that Waardenburg syndrome is caused by mutations of *PAX3* and other genes such as *MITF*, *SOX10*, *EDN3*, *EDNRB* and *SNAI2*^1,9,13,35–37^. Approximately 70% of Waardenburg syndrome patients suffer from sensorineural hearing loss through life and *Pax3* is the most common causative mutation for Waardenburg syndrome (type 1 and 3)^18^. Here, we used a mouse model of Pax3 deficiency and found that loss of *Pax3* causes a reduction of melanocytes in the developing cochlea, possibly stemming from a disruption to the distribution of neuroepithelial cells including neural crest cells. We showed that *Pax3*^*Cre/*+^ heterozygous mice had normal cochlear development and no hearing loss, while *Pax3*^*Cre/Cre*^ homozygous mice showed fewer cochlear melanocytes (intermediate cells) which are required for normal hearing. Although the *Pax3* knockout mice do not fully phenocopy Waardenburg syndrome in human, our and others’ results^6,23^ suggest that disruption of cochlear melanocytes as a result of Pax3 deficiency may contribute to their hearing loss. As a case in point, homozygous *PAX3* mutations have been reported in type 3 Waardenburg syndrome patients^15–17^.

Sensory epithelial cells in the inner ear are mostly derived from the otic vesicle^5,30^. Neuroepithelial cells including neural crest cells have also been proposed to contribute to the otic vesicle and later the sensory epithelium^5^. Neural crest cells detached from the neural tube ectoderm migrate to the otic vesicle and differentiate into various cell types such as melanocytes, Schwann cells and satellite cells in the developing cochlea^38^. Humans carrying *PAX3* mutations have abnormal development of melanocytes, manifested as heterochromia^1^. They also present with profound hearing loss despite grossly radiographically normal inner ear structures^24^. Previously, *Pax3* mutants including *Sp*, *Sp*^*2H*^ and *Pax3-Cre* mice have been analyzed for cochlear development and those heterozygous mice are identified by the presence of patchy pigmentation of skin hair, which is one of the major phenotypes of type 1 and 3 Waardenburg syndrome patients^5,6,9,10,12,23,27^. Although type 1 and 3 Waardenburg syndrome patients with *Pax3* heterozygous mutation typically exhibit severe-to-profound hearing loss, *Sp* heterozygous mice display normal hearing as do *Pax3-Cre* heterozygous mice in this study (Fig. 1C)^18,27^. Thus, the phenotype of heterozygous *Pax3-Cre* mice is less severe than in *Pax3*-heterozygous humans. In addition, we show that loss of *Pax3* causes shortened cochlea and malformed vestibular apparatus using *Pax3*^*Cre/Cre*^ homozygous mice which are inserted with the Cre recombinase cDNA followed by a stop codon and a polyA signal in *Pax3* exon 1, while having cochlear structures such as the stria vascularis^25^. Our study stands in contrast to the results of previous work using *Sp*^*2H*^*/Sp*^*2H*^ homozygous embryo, in which 32 nucleotides deletion of *Pax3* exon 5 by irradiation causes a truncated protein of its C-terminal half and prevents the formation of stria vascularis in the late embryonic cochlea^23,39,40^. This difference may be because the *Sp*^*2H*^*/Sp*^*2H*^ homozygous mice still expressed some Pax3 protein whereas *Pax3*^*Cre/Cre*^ homozygous mice displayed no detectable Pax3 protein. Furthermore, *Pax3*^*Cre/Cre*^ homozygous embryos in our study represent two distinct severities of phenotype. These diversities in phenotype may also be reflected in the variable degrees of hearing loss in type 1 and 3 Waardenburg syndrome caused by various *PAX3* gene mutations^41–43^.

Neural crest cells with pluri-potent potential differentiate into the various cell types including melanocytes and play important roles in the development of various organs^2,25^. Melanocytes originating from neural crest cells migrate into specific locations within the skin and hair follicles, and to other sites including stria vascularis in the cochlea^44^. Cochlear melanocytes are known as intermediate cells which generate high concentration of potassium ions in the cochlear endolymph^20,45^. Endocochlear potential in-turn drives depolarization of hair cells and is required for hearing function^46^. One may hypothesize that the hearing loss associated with Waardenburg syndrome is a result of a disruption of the endocochlear potential arising from the developmental disorder of cochlear melanocytes, among other factors^6,27^. In this study, we demonstrated that *Pax3* is necessary for the development of a full complement of cochlear melanocytes, with Pax3 deficiency leading to a reduction of cochlear melanocytes still expressing S100, *Dct* and *Kir4.1* in the stria vascularis. Thus, our study pointed out that a small number of cochlear melanocytes can still develop despite Pax3 deficiency, suggesting the presence of alternative regulators of differentiation of *Pax3*^+^ derivatives. During development, Schwann cell precursors migrate into the stria vascularis starting at around E15.5 to give rise to cochlear melanocytes^8^. Interestingly, a previous report using *Pax3-Cre* knock-in mice^25^ found no *Pax3*^+^ derivatives or *Dct*^+^ melanocytes in the stria vascularis at E15.5, although the distribution of *Pax3*^+^ derivatives remained normal in the glial cell region^6^. With the same mouse line as the above report, we observed *Pax3*^+^ derivatives in both the glial cell region and stria vascularis, and *Dct*^+^ melanocytes in the stria vascularis in the late embryonic period (E18.5). These divergent findings may be indicative of a delayed migration of *Pax3*^+^ derived cells from the glial region to other domains within the cochlea.

Finally, our data exhibited a degree of variance in the number of cochlear melanocytes in the stria vascularis caused by loss of *Pax3*. Whether this variance is indicative of the variable degree of hearing loss in Waardenburg syndrome remains unclear, as neither *Pax3* heterozygous or homozygous mice phenocopy Waardenburg syndrome patients^41–43^. As such, a mouse model (e.g. *Pax3* hypomorph) that more accurately models human Waardenburg syndrome is needed. The developing melanocytes in the human cochlea are considered as the prime target cells of gene therapy for Waardenburg syndrome^37^. Our results indicate that neuroepithelial cells with loss of *Pax3* can differentiate as melanocytes if they properly migrate into the stria vascularis. In conclusion, our data would guide future studies to develop hearing therapies for Waardenburg syndrome.

## Methods

### Mice

The following mouse strains were used: *Pax3*^*Cre/*+^ (Stock #005549, Jackson Laboratory)^25^, *CAG*^*CAT-EGFP/*+^ (gift from J. Miyazaki, Osaka Univ.)^47^, *R26R*^*mTmG*^ mice (stock #007576, Jackson Laboratory)^48^. Mouse embryos of both genders were used. Institutional Animal Care and Use Committee of The Jikei University School of Medicine (protocol number: 21-025, 2020-060) and Stanford University School of Medicine (protocol number: 18606) approved all procedures. All experimental procedures were performed in accordance with relevant guidelines and regulations. This study is reported in accordance with ARRIVE guidelines, https://arriveguidelines.org.

### Genotyping

Mouse genomic DNA was isolated from collected tail tips by adding 180 μl of 50 mM NaOH and incubating at 98 °C for 10 min, followed by the addition of 20 μl of 1 M Tris–HCl. PCR was performed to genotype transgenic mice with three specific primers which sequences were described in a previous paper^25^.

### Auditory physiology measurements

Auditory brainstem responses were recorded as described in a previous paper^49^. Briefly, P56 mice were anesthetized with a ketamine/xylazine mixture (100 mg/kg ketamine and 10 mg/kg xylazine, IP) and placed on a heating pad at 37 °C. Auditory brain responses were measured with a needle electrode which was located inferior to the tympanic bulla, referenced to an electrode on the vertex of the head, and a ground electrode was inserted at the hind limb. Tone burst stimuli were delivered with frequencies ranging from 8 to 32 kHz (8.0, 16.0, 32.0 kHz) up to 90 dB sound pressure level (SPL) in 5 dB steps. At each frequency and SPL, 512 trials were tested and averaged. Two-way analysis of variance (ANOVA) with Sidak’s multiple comparisons test was used for comparison of ABR thresholds.

### H&E staining

P43 mice were deeply anesthetized with pentobarbital sodium (50 mg/kg, IP), and perfused transcardially with 4% paraformaldehyde (PFA) in 0.1 M phosphate-buffered saline (PBS), pH 7.4. Temporal bones were harvested and additionally fixed in 4% PFA overnight at 4 °C. After PBS wash, inner ears were dissected from temporal bones and decalcified in 0.125 M EDTA for 2 weeks. Inner ears were dehydrated, embedded in paraffin wax, and sectioned to 4 µm using microtome REM-710 (YAMATO). Then, sections were stained with Hematoxylin and Eosin.

### Immunohistochemistry

Methods were modified as previously reported^50,51^. Briefly, E11.5 and E18.5 heads were harvested and fixed in 4% PFA overnight at 4 °C and then embedded in Tissue Tek OCT compound (Sakura, Tokyo, Japan) and frozen. Sections (10 μm thickness) were prepared using a cryostat CM3050S (Leica). P1 whole mount cochleae were dissected and fixed in 4% PFA for 1 h at room temperature (RT). Tissues were permeabilized with 0.5% TritonX-100 in PBS for 1 h at RT, and then blocked with 10% goat or donkey serum, 0.1% TritonX-100 and 1% bovine serum albumin in PBS for 30 min at RT. The following primary antibodies were used: chicken anti-GFP (1:1,000, GFP-1020, Aves labs), rabbit anti-Myosin7a (1:1,000; Proteus Bioscience), goat anti-Kcnq1 (1:100, sc-10646, Santa Cruz Biotechnology), mouse anti-Pax3 (1:1,000, Developmental Studies Hybridoma Bank), rabbit anti-S100 (ready-to-use liquid, GA504, Dako) and goat anti-Sox2 (1:200, sc-17320, Santa Cruz Biotechnology). Primary antibodies were applied overnight in a humidified chamber at 4 °C. The following day, tissues were washed with PBS three times at 5 min intervals and then incubated with Alexa Fluor secondary antibodies (488, 546 or 647, 1:500, Invitrogen or Jackson ImmunoResearch) which were diluted in PBS containing 0.1% TritonX-100 and 1% bovine serum albumin for 2 h at RT. DAPI (1:10,000, Invitrogen) was also used for nuclear staining.

### In situ hybridization

Harvested E18.5 heads were fixed in 4% PFA overnight at 4 °C, embedded for cryosections and sliced into 10 μm sections as described above. RNA probe synthesis and section in situ hybridization were performed as previously described with some modifications^51^. Briefly, a digoxigenin-labeled antisense RNA probe was synthesized using the DIG RNA Labeling Kit (Promega) with plasmids containing the following mouse genes: *Dct* (forward primer, TCCCGAGGCAACCAACATCT; reverse primer, CAGTAGGGCAACGCAAAGGA) and *Kir4.1* (forward primer, GGACAAACCCTTATCTGATTCCA; reverse primer, TGCGCAATAAGAAGCACGAT). Slides were permeabilized with protein K (Roche, 5 μg/ml) in PBS with 0.1% Tween 20 for 10 min at 37 °C, incubated with 1 μg/ml digoxigenin-labeled riboprobe in hybridization buffer for 16 h at 70 °C, blocked with 10% heat-inactivated sheep serum in Tris-buffered saline containing 0.1% Tween 20 for 30 min at RT, incubated with anti-digoxigenin antibody-conjugated alkaline phosphatase (1:2,000, Roche) in Tris-buffered saline containing 0.1% Tween 20 and 1% heat-inactivated sheep serum for 2 h at RT. Antibody detection was performed by incubating slides with 0.2% nitroblue tetrazolium and 0.2% 5-Bromo-4-chloro-3-indolyl phosphate p-toluidine salt in detection solution (0.1 M NaCl, 0.1 M Tris–HCl [pH 9.5], 50 mM MgCl2, 1% Tween 20) for 40–48 h at RT.

### Imaging and cell quantification

Section cochleae were captured using Axio Imager D1 (Zeiss) for bright field images or LSM500/880 (Zeiss) for fluorescent images. Image analyses were performed using Zen Software (Zeiss) and Photoshop CS6 (Adobe Systems). Cells were quantified from each turn (apical, mid-apical, mid-basal and basal turn) in the developing stria vascularis of section images. Melanocytes in the stria vascularis were identified as *Dct*^+^ or *Kir4.1*^+^ cells immediately next to marginal cells.

### Statistical analyses

Statistical analyses were performed using Microsoft Excel (Microsoft) and GraphPad Prism 7.03 (GraphPad). Two-way ANOVA was used for comparison with two independent variables. P < 0.05 was considered statistically significant.

### Paint injection

Paint-filling of the inner ear was performed as previously described^30^. E15.5 mouse embryos were harvested and fixed overnight in Bodian’s fixative. Samples were then dehydrated with ethanol and cleared with methyl salicylate. Glass micropipette was inserted in the utricle, and then inner ears were visualized by injecting white latex paint in 0.1% methyl salicylate into the membranous labyrinth. Samples were captured with stereomicroscope SZ61 (Olympus).

### Morphological picture

Harvested E18.5 mouse embryos were captured and then temporal bones were harvested and fixed in 4% PFA overnight at 4 °C. Following several PBS washes, inner ears were isolated from temporal bones including cochlear capsule in PBS. Samples were captured with stereomicroscope Lumar.V12 (Zeiss).

### Supplementary Information

Supplementary Information.

## Data Availability

All data analyzed in this study are included in this article and its supplementary information files. Mouse nucleotide sequences of *Dct* (Accession Number: NM_010024.3) and *Kir4.1* (Accession Number: NM_001039484.1) genes were referenced from the National Institutes of Health (NIH) genetic sequence database GenBank, https://www.ncbi.nlm.nih.gov/genbank/.
