# Supplementary material for: Loss of Pax3 causes reduction of melanocytes in the developing mouse cochlea
Source: Sci Rep. 2024 Jan 26;14:2210. doi: 10.1038/s41598-024-52629-9 (PMC10817906; doi:10.1038/s41598-024-52629-9)
Supplement: Supplementary file 1 — Supplementary Information. [file 41598_2024_52629_MOESM1_ESM.pdf]

Supplementary Materials

Loss of Pax3 causes reduction of melanocytes in the developing mouse cochlea

Tomokatsu Udagawa[1,2,3,†,*], Erisa Takahashi[1,2,†], Norifumi Tatsumi[2], Hideki Mutai[4], Hiroki Saijo[2], Yuko Kondo[1], Patrick J. Atkinson[5], Tatsuo Matsunaga[4], Mamoru Yoshikawa[3], Hiromi Kojima[1], Masataka Okabe[2], Alan G. Cheng[5]

[1]Department of Otorhinolaryngology, The Jikei University School of Medicine, Tokyo, Japan
[2]Department of Anatomy, The Jikei University School of Medicine, Tokyo, Japan
[3]Department of Otorhinolaryngology, Toho University School of Medicine, Tokyo, Japan
[4]Division Hearing and Balance Research, National Institute of Sensory Organs, NHO Tokyo Medical Center, Tokyo, Japan
[5]Department of Otolaryngology-Head and Neck Surgery, Stanford University School of Medicine, Stanford, CA, 94305, USA

[†]These authors contributed equally to this work

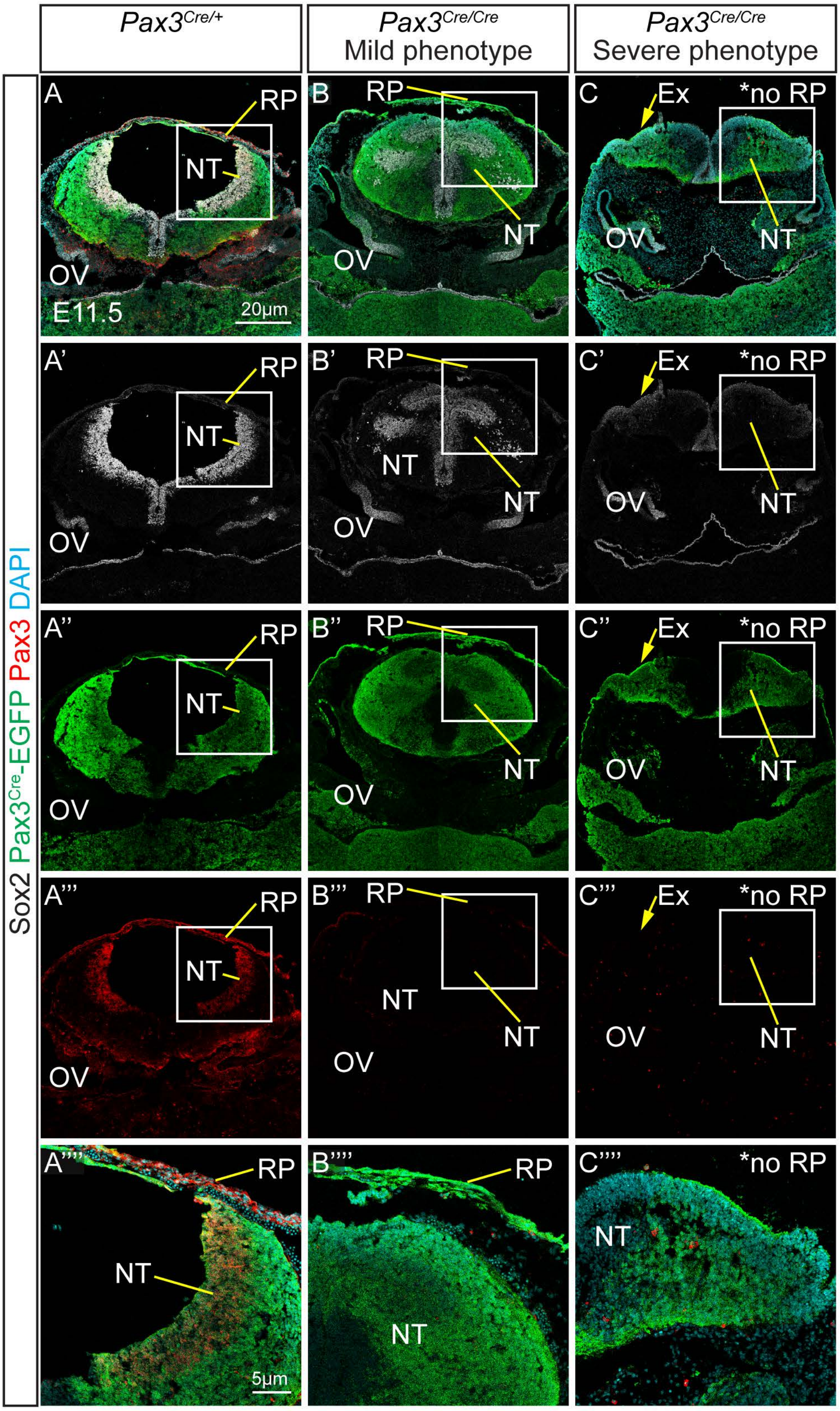

Supplementary Figure 1. Lack of Pax3 expression in the *Pax3* knockout mice. (A-A'''') *Pax3*$^{+}$ derivatives were detected in the brain (neural tube) and roof plate. Pax3 was expressed in both Sox2^{+} Pax3^{Cre}-EGFP^{+} epithelial layer of the brain and a subset of roof plate cells of E11.5 *Pax3*$^{Cre/+}$ heterozygous mice. (B-B'''', C-C'''') E11.5 *Pax3*$^{Cre/Cre}$ homozygous mice with a mild or severe phenotype did not display Pax3 expression. Mutants with a severe phenotype lacked the roof plate (asterisk). Ex, exencephaly; NT, neural tube; OV, otic vesicle; RP, roof plate.

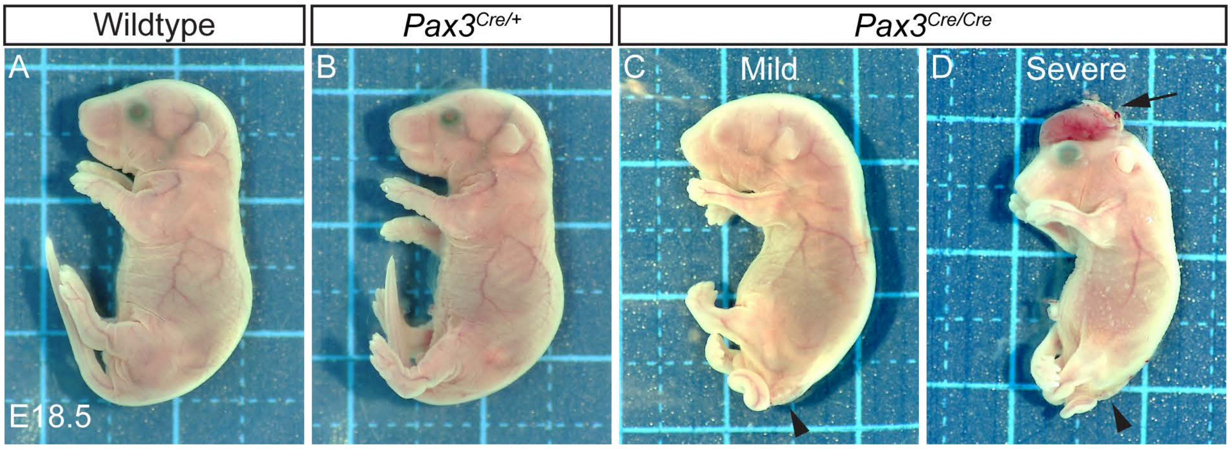

Supplementary Figure 2. *Pax3* knockout body phenotypes at late embryonic age. (A-B) Body shapes and sizes of wildtype and the *Pax3*$^{Cre/+}$ heterozygous embryos were similar at E18.5. (C) Mild phenotype of the E18.5 *Pax3*$^{Cre/Cre}$ homozygous embryo displayed spina bifida (arrowhead) and hypopigmentation of the eyes. (D) Severe phenotype of the E18.5 *Pax3*$^{Cre/Cre}$ homozygous embryo exhibited small body, exencephaly (arrow), and spina bifida (arrowhead).

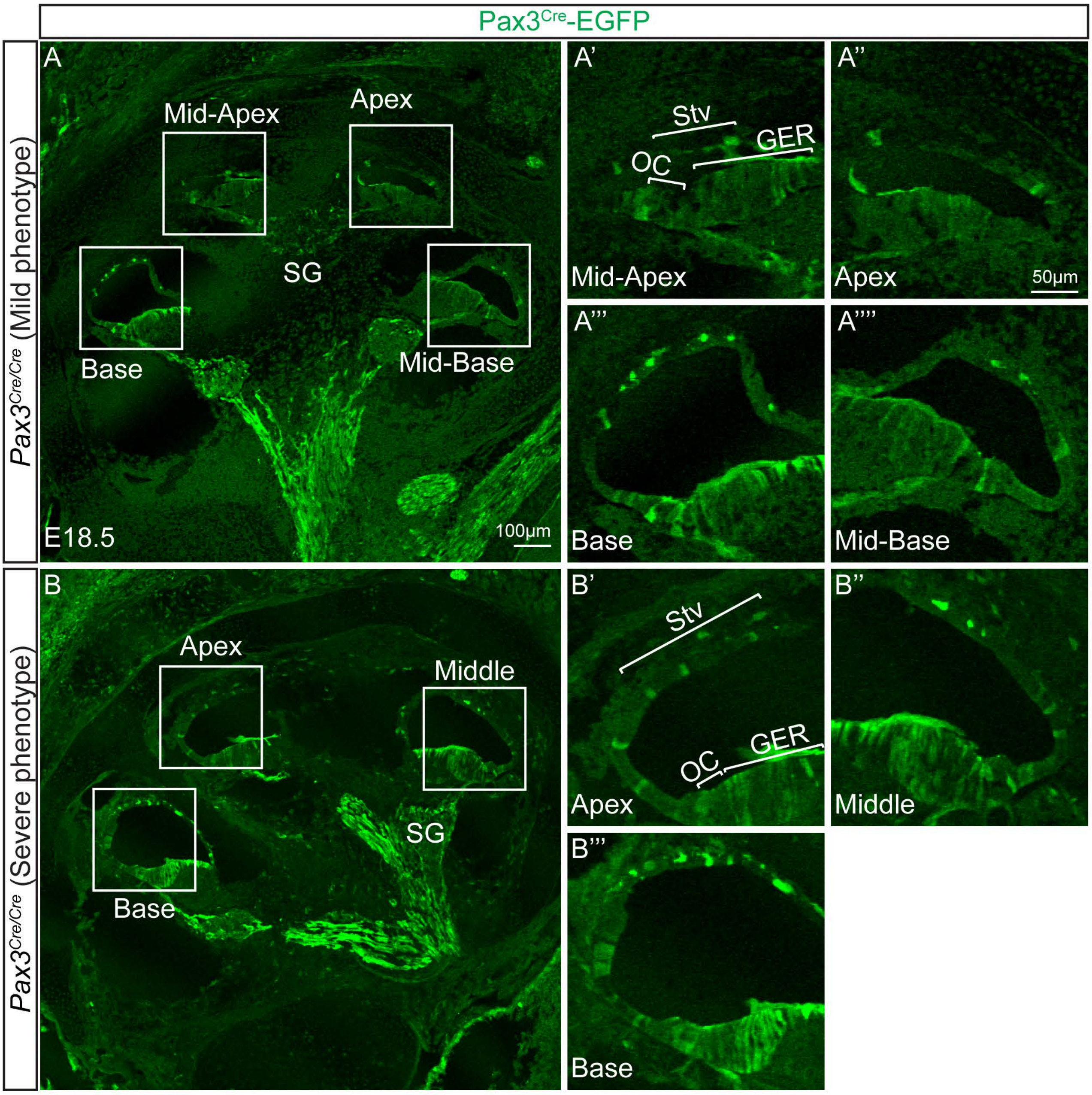

Supplementary Figure 3. Lineage tracing of neuroepithelial cells in the *Pax3* knockout cochleae. (A-A'''', B-B''') In the stria vascularis of the E18.5 *Pax3*$^{Cre/Cre}$ cochlea with both mild and severe phenotypes, there were a few Pax3^{Cre}-EGFP^{+} cells in each cochlear turn. GER, greater epithelial ridge; OC, organ of Corti; SG, spiral ganglion; StV, stria vascularis.

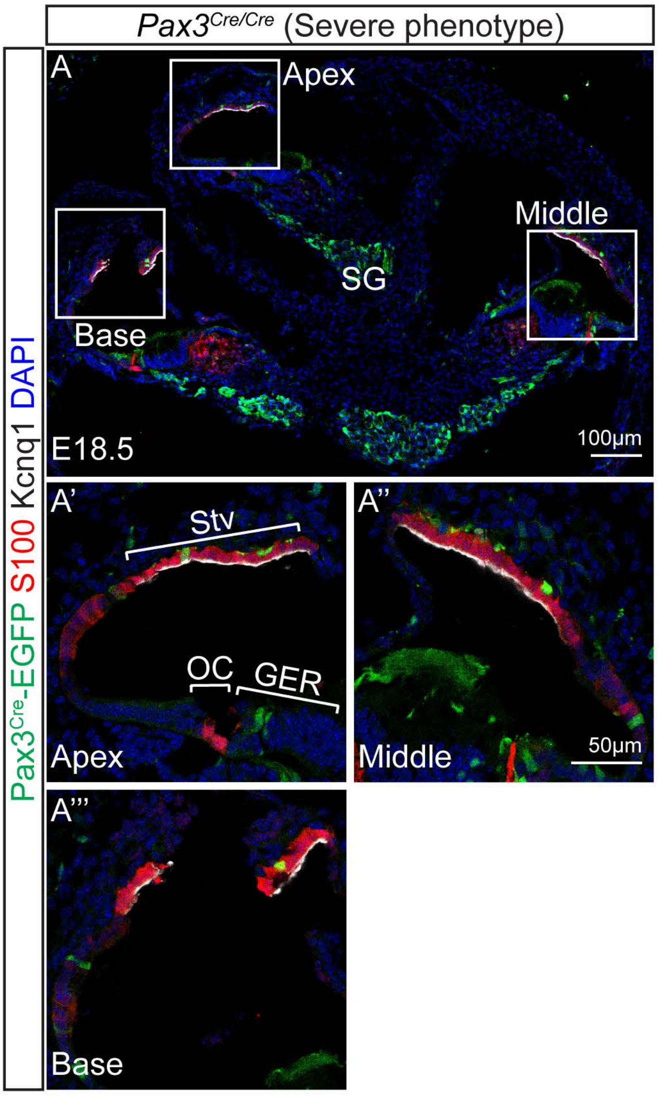

Supplementary Figure 4. Reduced intermediate cells in the *Pax3* knockout cochlea with a severe phenotype. (A-A''') In the stria vascularis of the E18.5 *Pax3*$^{Cre/Cre}$ cochlea with a severe phenotype, rare S100^{+} Pax3^{Cre}-EGFP^{+} intermediate cells were found next to Kcnq1^{+} S100^{+} marginal cells. GER, greater epithelial ridge; OC, organ of Corti; SG, spiral ganglion; StV, stria vascularis.

Supplementary Table S1. Genotypes and body phenotypes of E18.5 offspring from *Pax3*$^{Cre/+}$ heterozygous intercrosses

Genotype	+/+	Cre/+	Cre/Cre	Total
number of embryos	27	37	14	78

Body phenotype	+/+	Cre/+	Cre/Cre		Total
			Mild	Severe	
No Loop tail with Spina bifida or Exencephaly	27	37	1	0	65
Only Exencephaly	0	0	0	0	0
Only Loop tail with Spina bifida	0	0	7	0	7
Both Loop tail with Spina bifida and Exencephaly	0	0	0	6	6
